# Normativity and health promotion across biopsychosocial ecosystems

**DOI:** 10.3389/fpsyg.2025.1570813

**Published:** 2025-04-25

**Authors:** Federica Mauro, Paul Greenman, Michela Di Trani

**Affiliations:** ^1^Faculty of Medicine and Psychology, Sapienza University of Rome, Rome, Italy; ^2^Sapienza University of Rome, Rome, Italy; ^3^Département de Psychoéducation et de Psychologie, Université du Québéc en Outaouais, Gatineau, QC, Canada

**Keywords:** normativity (Canguilhem), health psychology, wellbeing, public health, health, collective health

## Introduction

Over the years, there have been many definitions of “health psychology,” each of which represents an attempt to delineate the boundaries of this complex and multifaceted field (see Table 1).

**Table 1:** Health psychology's definitions.

**Author**	**Definition**
Matarazzo (1982)	“Health Psychology is the aggregate of the specific educational, scientific, and professional contributions of the discipline of psychology to the promotion and maintenance of health, the prevention and treatment of illness, the identification of etiologic and diagnostic correlates of health, illness, and related dysfunction and to the analysis and improvement of the health care system and health policy formation.”
Johnston (1994)	“Health psychology is defined as the study of psychological and behavioral processes in health, illness, and health care.”
Kaholokula and Haynes (2015)	“Health psychology and behavioral medicine are interdisciplinary fields devoted to developing and integrating the biomedical, behavioral, psychological, and social sciences and approaches to better understand health and illness, and to applying these sciences and approaches to prevention, diagnosis, treatment, and rehabilitation.”
Crombez et al. (2022)	“Health psychology is a rich and diverse field within psychology. It is not easy to capture its key principles in a few sentences. Nevertheless, let me provide you some of my guiding principles, as follows: (1) Health psychology is about a normal psychology of human behavior, cognition, and emotion. It is largely about normal individuals, like you and me, who may become trapped into abnormal contexts (e.g., cancer, diabetes, acquired brain injury, pain, and fatigue). We should therefore be cautious in applying concepts and models stemming from abnormal psychology (Crombez et al., 2020). (2) Health psychology reaches out to medical and other health-care professionals, and is par excellence a discipline with strong interdisciplinary and multidisciplinary collaborations. (3) Health psychologists do not only require knowledge and competences within the field psychology but also profound knowledge of other disciplines, such as medicine, nursing, and physiotherapy.”
Marks (2024)	“Health psychology is an interdisciplinary field concerned with the application of psychological knowledge and techniques to health, illness and health care.”

Despite some differences, all definitions emphasize the intricate relationship between psychological processes and physical health. Interventions in the realm of health psychology generally involve holistic and multifaceted strategies to address a wide range of health challenges. Integrating mind and body involves understanding how psychological and social factors influence physical health and illness, thereby enabling practitioners to develop more effective interventions. Collective health, on the other hand, can be viewed as both a scientific field that produces various forms of knowledge about the concept of health, and a “social space” (Vieira-da-Silva and Pinell, 2014; Silva et al., 2019) where practices involving actions are carried out across different organizations and institutions. This field has evolved to adopt an interdisciplinary approach that transcends the biomedical and pathology paradigms. Collective health embraces a broader perspective, considering material conditions and social factors that shape health outcomes. Its basic disciplines are epidemiology, health planning/management, and social sciences in health. Collective health provides a framework for understanding how social determinants, such as income inequality, education, and access to healthcare, affect health at a population level (Marmot and Wilkinson, 2006).

Within the framework of collective health, health psychology represents a comprehensive approach that bridges individual psychological processes with biological and broader social determinants of health. Whereas collective health highlights the structural and systemic factors that shape population-level outcomes, health psychology focuses on understanding the psychological and behavioral mechanisms that influence health. This integration stresses the necessity of addressing health challenges at multiple levels: individual, community, and social. To meet this need, in the course of time, distinct areas have arisen in the field of health psychology, including clinical health psychology, public health psychology, community health psychology, cultural health psychology, and critical health psychology (Marks, 2002; Hatala, 2012). This variety of approaches highlights the importance of adapting theories and application models to specific contexts and needs, while maintaining a unifying paradigm for health that provides coherence and allows different theories and models to derive meaning and interconnect (Marks et al., 2024).

## Health psychology's theoretical frameworks

In 1946, the World Health Organization (WHO) defined health as “a state of complete physical, social, and spiritual wellbeing, not merely the absence of illness.” This definition emphasizes the multidimensional nature of health and acknowledges the need to integrate psychological and social dimensions alongside physical wellbeing.

Aligned with this broader perspective, Engel (1977) introduced the Biopsychosocial Model (BPSM) to address the shortcomings of the traditional biomedical model. Engel proposed that all natural phenomena exist within a hierarchy of interconnected systems, ranging from the biosphere at one end to societal and individual behavior in the middle and down to the cellular and subatomic levels. He argued that understanding health and illness requires considering these interrelated levels, focusing on the interaction among biological, psychological, and social factors. While not without critics (e.g., Hatala, 2012; Marks et al., 2024), BPSM is largely considered a cornerstone of health psychology. As a holistic framework for understanding the complexities of health and disease, it marks a deliberate effort within the field to challenge the dominance of the biomedical model while highlighting psychology's critical role in addressing health and illness comprehensively, integrating various dimensions of human experience (Murray, 2014).

A more general multi-level framework has since been proposed, which synthesizes both the biological determinants and the social context of health-related experience and behavior (Marks, 1996; Marks et al., 2024). This offers a broader perspective than the narrow focus of traditional psychological models of health, recognizing the variable nature of health and the need for a multidisciplinary approach. The “onion model,” adapted from Dahlgren and Whitehead (1991), is centered on the individual, characterized by fixed factors such as age, sex, and genetic predispositions. Four layers of influence and mechanisms surround this core and are assumed to bring about change: individual lifestyle; social and community factors; living and working conditions; and broader socioeconomic, cultural, and environmental contexts.

In light of these frameworks, we may define the interconnected system of biological, psychological, social, cultural, and environmental dimensions that determine the health of an individual as a “biopsychosocial ecosystem.” This concept borrows from the biological definition of an ecosystem (Millennium Ecosystem Assessment, 2005), highlighting the interdependence and reciprocal relationships between various components. Health, wellbeing, and human behavior emerge from the dynamic interplay of biological elements (genetics, physiology), psychological processes (thoughts, emotions, behaviors), social influences (relationships, cultural norms, policies), and environmental influences. This term emphasizes how those components do not operate in isolation; rather, they are part of an integrated system where changes or disruptions in one domain (e.g., stress, medical treatments) can affect others (e.g., physical health and social relationships). From this perspective, such integration creates a holistic biopsychosocial ecosystem that shapes the individual's overall functioning, health, wellbeing, and capacity to adapt to challenges.

## Health, wellbeing and their relationship

Defining health and wellbeing remains a complex challenge (Christoforou et al., 2024). Beyond the WHO's definition, two primary concepts of health have been identified: a biomedical perspective, which views health as the absence of abnormalities, and a functional perspective, which describes health as “the strength to be” and the ability to overcome challenges (Misselbrook, 2014). Broader interpretations of health also emphasize fulfilling social roles, maintaining independence, fostering optimism, and, in some cases, integrating spirituality. Moreover, both internal factors, such as personal adaptation, and external elements, including socio-economic and environmental policies, have been considered essential components of health (Christoforou et al., 2024).

Similarly, defining wellbeing poses challenges. Wellbeing is a multifaceted construct with definitions that vary by domain, often described as a more emotional and social concept than a health-related one, encompassing positive feelings, effective functioning, and contributions to society (Bautista et al., 2023).

A related concept is quality of life (QoL), which refers to an individual's perception of their life circumstances, shaped by cultural context, personal goals, and expectations, and influenced by physical health, psychological wellbeing, independence, social relationships, and the environment (Whoqol Group, 1995). It overlaps with subjective wellbeing (SWB) (Marks et al., 2024), which encompasses evaluations of life, emotional states, and a sense of meaning and purpose. SWB, as defined by Diener (2006) and Durand (2015), includes life evaluation (e.g., joy, pride), current affect (e.g., emotions like anger or worry), and eudaemonia (a sense of purpose).

The interconnection between health and wellbeing is frequently discussed, with some definitions, such as the WHO's, including wellbeing as an integral part of health, and strong evidence has linked higher SWB to better health outcomes and longer life, highlighting its critical role in wellbeing (Marks et al., 2024).

However, definitions of health and wellbeing differ across disciplines, cultures, and individuals and are shaped by personal characteristics and needs. This subjectivity complicates efforts to establish a general definition, which, however, is needed (Davies, 2009). While some studies focus on defining these concepts for specific age groups, an interdisciplinary approach addressing risk and resilience factors may provide a more inclusive foundation for defining health and wellbeing across diverse populations (Marks, 2024).

Going toward this direction, homeostasis theories of wellbeing (Marks, 2024) were proposed to understand the links between subjective wellbeing and health, offering theoretical frameworks that conceptualize wellbeing as a dynamic state of equilibrium within an individual. Drawing from the biological principle of homeostasis, referred to as the body's ability to maintain internal stability despite external changes, these models suggest that individuals strive to maintain a stable level of wellbeing through adaptive processes, offering descriptions of the dynamic interplay between subjective wellbeing, health, and contextual factors.

## Normativity and the definition of health

As shown in Table 1, the concept of illness rooted in the traditional biomedical model still plays a central role in defining health psychology. Within this view, health is equated with statistical “normality,” and illness is seen as a deviation from the norm (Braibanti, 2015).

However, Canguilhem (1966) proposed a broader understanding of “norm,” emphasizing the relationship between the organism and its environment. Health, in this perspective, is not the absence of disease but the ability to create and adapt to new norms. The pathological is not the opposite of the normal, but a different mode of normativity—revealing how illness alters the quality of life by limiting interaction with the environment and imposing new rhythms. Medicine, then, is rooted in the lived experience of disruption caused by illness.

Health becomes less about fixed states and more about normative flexibility—life's capacity to adapt creatively. Even illness, while it restricts this creativity, is part of a broader process of adaptation. In extreme cases, such as prolonged hospitalization, this process can stagnate, leading to a monotony that mirrors the loss of vitality.

This view of normativity underscores the dynamic regulation of human functioning within biopsychosocial ecosystems, framing health not as a static outcome but as a salutogenic process. Adaptability and experiential richness are essential for individuals to navigate life meaningfully—like a wanderer shaping their path through the challenges of existence (Bertini, 2012).

In line with this, a recent expansion of the psychological wellbeing model introduces a third dimension—psychological richness, which highlights the value of diverse, meaningful experiences and adaptability in human development (Oishi et al., 2021; Mauro et al., 2025).

## The role of health promotion and future perspectives

The conceptualization of health as a salutogenic process, rather than merely the absence of disease, highlights the critical role of health psychology and, more specifically, health promotion within the framework of collective health. Defining health as an ongoing process rather than a fixed outcome offers a unified understanding of health and wellbeing. This may, moreover, facilitate the integration of health psychology practices across diverse contexts, addressing the specific needs and challenges present within various biopsychosocial ecosystems.

This proposed framework is moreover grounded in the principles of Patient-Centered Care (PCC), a foundational model in contemporary healthcare that recognizes the patient as a whole person, taking into account not only biomedical aspects but also psychological, social, and existential dimensions. At its core, PCC is based on building a therapeutic alliance that honors the patient's values, needs, preferences, and autonomy (Mead and Bower, 2000), emphasizing that “*the patient's experience of illness should be the starting point of clinical practice”* (Stewart et al., 2014).

PCC has been associated with improved clinical outcomes, higher patient satisfaction, increased treatment adherence, and reduced healthcare costs (Epstein and Street, 2011). Furthermore, it aligns with the ethical principles of autonomy, beneficence, and respect.

According to the World Health Organization (2007), health promotion empowers individuals to gain greater control over and improve their health. Unlike disease prevention, which targets specific conditions, or health education, which aims to inform, health promotion addresses both individual and contextual factors shaping behavior. It seeks not only to prevent illness but also to enhance overall wellbeing by equipping individuals with the resources and skills necessary to actively manage and shape their health (World Health Organization, 2007; Nutbeam, 2000; Kickbusch, 1995).

This holistic approach, integrating physical, mental, and social dimensions of health, fosters resilience and quality of life, highlighting the transformative potential of health promotion in advancing global public health goals (Marks et al., 2024). As such, it becomes essential to embed health promotion practices within all settings—beginning with healthcare systems and expanding to schools, workplaces, and communities. As Marks (2024) notes, diverse tools and strategies are required within health psychology to address the specific needs of various contexts. Despite their differences, these approaches can be unified under the view of health and wellbeing as normative processes, shifting the focus from disease causation to strengthening individuals' capacity to pursue and sustain health (Antonovsky, 1996). The integration of quantitative, qualitative, and participatory research methods within this paradigm allows for a more comprehensive understanding of health as a lived, dynamic, and context-sensitive experience.

## References

[B1] Antonovsky A. (1996). The salutogenic model as a theory to guide health promotion. Health Promot. Int. 11, 11–18. 10.1093/heapro/11.1.11

[B2] Bautista T. G. Roman G. Khan M. Lee M. Sahbaz S. Duthely L. M.. (2023). What is wellbeing? A scoping review of the conceptual and operational definitions of occupational wellbeing. J. Clin.Transl. Sci. 7:e227. 10.1017/cts.2023.648 38028344 PMC10643923

[B3] Bertini M. (2012). Il senso della salute: Percorsi e prospettive in psicologia della salute. Milan: FrancoAngeli.

[B4] Braibanti P. (2015). Ripensare la salute: per un riposizionamento critico nella psicologia della salute. Milan: FrancoAngeli.

[B5] Canguilhem G. (1966). Le Normal et le Pathologique. Paris: Presses Universitaires de France.

[B6] Christoforou R. Lange S. Schweiker M. (2024). Individual differences in the definitions of health and wellbeing and the underlying promotional effect of the built environment. J Build. Eng. 84:108560. 10.1016/j.jobe.2024.108560

[B7] Crombez G. De Paepe A. L. Veirman E. Eccleston C. Verleysen G. Van Ryckeghem D. M. L. (2020). Let's talk about pain catastrophizing measures: an item content analysis. PeerJ, 8:e8643. 10.7717/peerj.8643 32181053 PMC7060750

[B8] Crombez Y. Ghyselen A.-S. Winter-Froemel E. Zenner E. (2022). The sociopragmatic parameters steering the reported selection of Anglicisms or their Dutch alternatives. Linguistics 60, 973–1010. 10.1515/ling-2020-0237

[B9] Dahlgren G. Whitehead M. (1991). Policies and strategies to promote social equity in health. Stockholm: Institute for Futures Studies.

[B10] Davies P. G. (2009). Why the definition of health matters. BMJ 338:b1437. 10.1136/bmj.b28 19136537

[B11] Diener E. (2006). Guidelines for national indicators of subjective wellbeing and ill-being. Appl. Res. Qual. Life 1, 151–157. 10.1007/s11482-006-9007-x

[B12] Durand M. (2015). The OECD better life initiative: how's life? and the measurement of wellbeing. Rev. Income Wealth 61, 4–17. 10.1111/roiw.12156

[B13] Engel G. L. (1977). The need for a new medical model: a challenge for biomedicine. Science. 196, 129–136. 10.1126/science.847460 847460

[B14] Epstein R. M. Street R. L. (2011). The values and value of patient-centered care. Ann. Family Med. 9, 100–103. 10.1370/afm.1239 PMC3056855 21403134

[B15] Hatala A. R. (2012). The status of the “biopsychosocial” model in health psychology: towards an integrated approach and a critique of cultural conceptions. Open J. Med. Psychol. 1, 51–62. 10.4236/ojmp.2012.14009

[B16] Johnston M. (1994). Health psychology: current trends and future directions. Psychol. Health 9, 407–420.

[B17] Kaholokula J. K. Haynes S. N. (2015). Health psychology and behavioral medicine: contributions to prevention and treatment of chronic conditions. Behav. Ther. 46, 167–183.

[B18] Kickbusch I. (1995). The contribution of the world health organization to a new public health and health promotion. Am. J. Public Health 85, 1297–1300. 10.2105/ajph.93.3.383 PMC1447748 12604477

[B19] Marks D. F. (1996). Health psychology in context. J. Health Psychol. 1, 7–21. 10.1177/135910539600100102 22011517

[B20] Marks D. F. (2002). Freedom, responsibility and power: contrasting approaches to health psychology. J. Health Psychol. 7, 5–19. 10.1177/1359105302007001062 22114223

[B21] Marks D. F. (2024). Homeostasis theory of wellbeing. J. Health Psychol. 29, 721–733. 10.1177/13591053231216014 38230535

[B22] Marks D. F. Murray M. Locke A. Annunziato R. A. Estacio E. V. (2024). Health Psychology: Theory, Research and Practice (6th ed.). SAGE Publications.

[B23] Marmot M. Wilkinson R. G. (2006). Social determinants of health (2nd ed.). Oxford: Oxford University Press.

[B24] Matarazzo J. D. (1982). Behavioral health and behavioral medicine: frontiers for a new health psychology. Am. Psychol. 37, 807–817. 10.1037/0003-066X.35.9.807 7416568

[B25] Mauro F. Di Trani M. Simione L. (2025). The psychologically rich life questionnaire: Italian validation and exploration of its relationships with mindfulness, self-compassion, and cognitive fusion within the health psychology framework. Front. Psychol. 16:1525300. 40051408 10.3389/fpsyg.2025.1525300 PMC11883556

[B26] Mead N. Bower P. (2000). Patient-centredness: a conceptual framework and review of the empirical literature. Soc. Sci. Med. 51, 1087–1110. 10.1016/S0277-9536(00)00098-8 11005395

[B27] Millennium Ecosystem Assessment (2005). Ecosystems and Human Well-being: Synthesis. Washington, DC: Island Press.

[B28] Misselbrook D. (2014). W is for well-being and the WHO definition of health. Br. J. Gen. Pract. 64:582. 10.3399/bjgp14X682381 25348979 PMC4220217

[B29] Murray M. (2014). Social history of health psychology: context and textbooks. Health Psychol. Rev. 8, 215–237. 10.1080/17437199.2012.701058 25053134

[B30] Nutbeam D. (2000). Health literacy as a public health goal: a challenge for contemporary health education and communication strategies into the 21st century. Health Promot. Int. 15, 259–267. 10.1093/heapro/15.3.259

[B31] Oishi S. Choi H. Liu A. Kurtz J. (2021). Experiences associated with psychological richness. Eur. J. Pers. 35, 739–755. 10.1177/0890207020962334

[B32] Silva M. J. D. S. Schraiber L. B. Mota A. (2019). The concept of health in Collective Health: contributions from social and historical critique of scientific production. Physis Revista de Saúde Coletiva 29:e290102. 10.1590/s0103-73312019290102

[B33] Stewart M. Brown J. B. Weston W. W. McWhinney I. R. McWilliam C. L. Freeman T. R. (2014). Patient-Centered Medicine: Transforming the Clinical Method, 3rd edn. Boca Raton, FL: CRC Press.

[B34] Vieira-da-Silva L. M. Pinell P. (2014). The genesis of collective health in Brazil. Sociol. Health Illn. 36, 432–446. 10.1111/1467-9566.12069 24111568

[B35] Whoqol Group (1995). The World Health Organization quality of life assessment (WHOQOL): position paper from the World Health Organization. Soc. Sci. Med., 41, 1403–1409. 10.1016/0277-9536(95)00112-K 8560308

[B36] World Health Organization (2007). The International Network of Health Promoting Hospitals and Health Services: integrating health promotion into hospitals and health services: concept, framework and organization (No. WHO/EURO: 2007-4838-44601-63250). World Health Organization. Regional Office for Europe.

